# Sirt3 Maintains Microvascular Endothelial Adherens Junction Integrity to Alleviate Sepsis-Induced Lung Inflammation by Modulating the Interaction of VE-Cadherin and *β*-Catenin

**DOI:** 10.1155/2021/8978795

**Published:** 2021-10-01

**Authors:** Dan-Qian Chen, Mei-Jia Shen, Hui Wang, Yan Li, A-Ling Tang, Shan Li, Meng-Chen Xiong, Yan Guo, Guo-Qiang Zhang

**Affiliations:** ^1^Department of Emergency, China-Japan Friendship Hospital, No. 2 Yinghua Dongjie, Beijing 100029, China; ^2^Department of Critical Care Medicine, Weifang Hospital of Traditional Chinese Medicine, No. 1055 Weizhou Road, Weifang, Shandong 261041, China; ^3^Department of Internal Medicine, University of New Mexico, 1700 Lomas Blvd. NE, Albuquerque, New Mexico 87131, USA

## Abstract

Inflammatory injury is a hallmark of sepsis-induced acute respiratory distress syndrome (ARDS)/acute lung injury (ALI). However, the mechanisms underlying inflammatory injury remain obscure. Here, we developed the novel strategy to suppress lung inflammation through maintaining microvascular endothelial barrier integrity. VE-cadherin is the main adherens junction protein that interacts with *β*-catenin and forms a complex. We found that lung inflammation was accompanied by decreased VE-cadherin expression and increased *β*-catenin activity in animal models and human pulmonary microvascular endothelial cells (HPMECs), illuminating the relationship among VE-cadherin/*β*-catenin complex, microvascular endothelial barrier integrity, and inflammation. Furthermore, we showed that the VE-cadherin/*β*-catenin complex dissociated upon lung inflammation, while Sirt3 promoted the stability of such a complex. Sirt3 was decreased during lung inflammation *in vivo* and *in vitro*. Sirt3 deficiency not only led to the downregulation of VE-cadherin but also enhanced the transcriptional activity of *β*-catenin that further increased *β*-catenin target gene MMP-7 expression, thereby promoting inflammatory factor COX-2 expression. Sirt3 overexpression promoted VE-cadherin expression, inhibited *β*-catenin transcriptional activity, strengthened the stability of the VE-cadherin/*β*-catenin complex, and suppressed inflammation in HPMECs. Notably, Sirt3 deficiency significantly damaged microvascular endothelial barrier integrity and intensified lung inflammation in animal model. These results demonstrated the role of Sirt3 in modulating microvascular endothelial barrier integrity to inhibit inflammation. Therefore, strategies that aim at enhancing the stability of endothelial VE-cadherin/*β*-catenin complex are potentially beneficial for preventing sepsis-induced lung inflammation.

## 1. Introduction

Sepsis-induced acute respiratory distress syndrome (ARDS)/acute lung injury (ALI) is characterized by severe hypoxemia and acute respiratory failure and results in pulmonary vascular injury, edema formation, and inflammation [1, 2]. Microvascular endothelial barrier dysfunction, the main pathophysiological feature of ARDS/ALI, induces capillary leakage and edema that further intensifies inflammatory injury, thus causing high morbidity and mortality [2]. Inflammatory mediators that target the adherens junction destroy microvascular endothelial cell junctions and promote vascular permeability [3]. Adherens junctions are composed of transmembrane proteins and form the interaction between adjacent cells, which maintains the integrity of the microvascular endothelial monolayer and modulates its barrier function. A single span transmembrane, the vascular endothelial cadherin (VE-cadherin) is the main adherens junction protein that is linked to the cortical F-actin cytoskeleton intracellularly in the vascular endothelial cell. Upon inflammatory injury, in a vicious circle, VE-cadherin undergoes phosphorylation, destabilization, and internalization that directly damages the microvascular endothelial barrier and intensifies inflammation [4, 5]. In the meantime, sepsis-induced inflammation contributes to the breakdown of the microvascular endothelial barrier that further amplifies inflammation [6].

As an intracellular scaffold protein, *β*-catenin interacts with VE-cadherin that not only regulates microvascular endothelial barrier integrity but also participates in inflammation [7, 8]. *β*-Catenin plays a significant role in ARDS/ALI. While the inhibition of *β*-catenin remarkably increases animal survival in the sepsis-induced ALI model [9], the activation of *β*-catenin contributes to inflammation through mediating the downstream target gene expression in ARDS/ALI [10, 11]. Once triggered by Wnt ligands, the *β*-catenin protein is stabilized and accumulated in the cytoplasm and further translocates into the nucleus where *β*-catenin binds to T-cell factor/lymphoid enhancer factor (TCF/LEF) to initiate downstream target gene expression, including matrix metallopeptidase-7 (MMP-7) [12, 13]. MMP-7 could increase permeability between endothelial cells [14]. Additionally, the activation of *β*-catenin also results in the upregulation of angiotension-2 protein, which damages microvascular endothelial barrier integrity [15, 16]. However, the role of *β*-catenin and VE-cadherin interaction in regulating microvascular endothelial barrier integrity remains unclear in ARDS/ALI that warrants further investigation.

The emerging roles of the Sirtuin (Sirt) family in sepsis-induced ARDS/ALI have attracted extensive attention [17–19]. The Sirt family belongs to the class III histone deacetylases that are composed of Sirt1-7. Sirt1 is a nuclear NAD^+^-dependent histone deacetylase, while Sirt3 is localized in the mitochondria. Previous studies have fully explored the role of Sirt1 in sepsis-induced ARDS/ALI, and they found that Sirt1 protects lung from inflammation [20–23]. Sirt1 also has been reported in maintaining vascular endothelial barrier integrity in lung tissue [20]. Sirt3, another member of the Sirt family, participates in mitochondrial oxidative stress, bioenergetics, apoptosis, mitochondrial fission, and metabolic reprogramming [24–26]. However, there is a lack of definite evidences elucidating the effects and underlying mechanisms of Sirt3 in microvascular endothelial barrier integrity. Therefore, the present study is aimed at investigating whether Sirt3 alleviates sepsis-induced inflammation by maintaining lung microvascular endothelial barrier integrity *via* the interaction of *β*-catenin and VE-cadherin.

## 2. Materials and Methods

### 2.1. Animal Treatment

The animal study was approved by the Ethics Committee of the Institute of Chinese Materia Medica, China Academy of Chinese Medical Sciences (No. 20,152,013), and was reported in compliance with the ARRIVE guidelines [27]. Male C57BL/6 mice, weighing 20-22 g, were purchased from the Laboratory Animal Center of the Academy of Military Medical Sciences (Beijing, China, Certificate No. SCXK 2002-0010) and randomly assigned to the experimental groups to establish lipopolysaccharide- (LPS-) and cecal ligation puncture- (CLP-) induced ALI models. The Sirt3^−/−^ mice were purchased from Cyagen Biosciences (Suzhou, China). Mice were intraperitoneally injected with sodium pentobarbital (1%, 50 mg/kg) for anesthesia. The complete CLP model was carried out by perforating the ligation end and squeezing out feces as previously described [28], while sham-operated mice were carried out with the same surgery without cecum ligation or puncture. Mice were administered LPS (20 mg/kg/day, *E. coli* 0111:B4, Sigma-Aldrich, USA) in sterile saline by intraperitoneal injection to induce ALI, while control mice were administrated sterile saline by intraperitoneal injection. Before CLP surgery or LPS injection, mice were administered 3-TYP (5 mg/kg/day, Selleck Chemicals, USA) or ICG-001 (5 mg/kg/day, Selleck Chemicals, USA) in sterile PBS by intraperitoneal injection for 2 days. Mice were sacrificed at 3 h, 6 h, and 24 h after CLP surgery or LPS injection. The lung tissues were immediately frozen and stored in liquid nitrogen for subsequent experiments.

### 2.2. Cytokine and Chemokine Measurements

Serum interleukin-6 (IL-6) and tumor necrosis factor-α (TNF-*α*) from LPS- or CLP-induced ALI mice were measured employing a commercially available ELISA kit and used according to the manufacturer's kit protocols (R&D Systems). Protein concentrations were measured spectrophotometrically at 450 nm and determined by establishing a standard curve by employing standard proteins.

### 2.3. Cell Culture and Treatment

Human pulmonary microvascular endothelial cells (HPMECs) were purchased from the China Center for Type Culture Collection. HPMECs were cultured in Endothelial Cell Medium (1001, ScienCell, USA) supplemented with 10% fetal bovine serum at 37°C with 5% carbon dioxide. HPMECs were treated with 0.5 ng/mL or 1.0 ng/mL LPS (*E. coli* 0111:B4, Sigma-Aldrich, USA). The concentrations of 3-TYP and ICG-001 were both 10 *μ*M. After 12 h or 24 h treatment, HPMEC cells were harvested for subsequent experiments.

### 2.4. Knock-In and Knockdown of Sirt3 *In Vitro*

The plasmid expressing full-length human Sirt3 cDNA (Sirt3 over), the plasmid containing empty plasmids (vector), the plasmid expressing shRNA against human Sirt3 (Sirt3 shRNA), and the plasmid containing scramble (scramble) were constructed by GeneChem (Shanghai, China). HPMECs were seeded in a 6-well plate with 70-80% confluence. The plasmid and Lipofectamine 3000 (Invitrogen, USA) were directly added to the medium, and the medium was removed after 24 h treatment according to the manufacturer's guide.

### 2.5. Histological Analysis

Briefly, lung tissues were fixed in 4% paraformaldehyde at room temperature for 24 h. After processing for paraffin embedding, lung tissues were sectioned at 5 *μ*m. Hematoxylin and eosin (HE) staining was performed by following the method routinely used in our laboratory [29]. The images were acquired using a Leica microscope (Leica Microsystems, Germany), and the lung injury score was assessed by five easily identifiable pathological processes according to the official ATS workshop report [30, 31].

### 2.6. Immunohistochemical Staining

Paraffin-embedded mouse lung sections and immunohistochemical staining for lung tissues were performed as described previously [32]. After heating in citrate buffer (10 mM sodium citrate, pH 6.0), the slides were blocked with 3% H_2_O_2_, incubated with 10% goat serum at room temperature for 1 h, and then incubated with primary antibodies at 4°C overnight. After incubating with secondary antibodies, the slides were developed by 3,3-N-diaminobenzidine tetrahydrochloride. The slides were then counterstained with Harris hematoxylin and mounted on neutral gum. The images were acquired with a Leica microscope (Leica Microsystems, Germany) and analyzed using Image-Pro Plus 6.0 software according to the mean optical density of positively stained areas in a standard protocol [32, 33]. To represent the results better, the value of protein expression in the sham-operated or control group was further normalized to 1 using the mean.

### 2.7. Immunofluorescence Staining and Confocal Microscopy

Briefly, HPMECs were cultured on coverslips and fixed with 4% paraformaldehyde at 4°C. The slides were incubated with 10% goat serum for 1 h and with primary antibodies at 4°C overnight and then incubated with secondary antibodies for 2 h and incubated with propidium iodide for 10 min. The slides were mounted with 80% glycerinum in sterile PBS and measured by a laser-scanning confocal microscope (Leica Microsystems, Germany). To clearly present the results, the value of protein expression in the control group was further normalized to 1 using the mean.

### 2.8. Western Blot Analysis

Protein expression was examined by Western blot as previously described [34]. HPMECs or lung tissues were lysed using RIPA buffer, and protein concentration was examined using the Pierce BCA protein assay kit (23227, Thermo Fisher Scientific, USA). The polyvinylidene difluoride (PVDF) membranes were purchased from GE Healthcare (10600023, USA). The following primary antibodies were employed: VE-cadherin (1 : 1000, ab33168, Abcam, USA), *β*-catenin (1 : 1000, 610154, BD Transduction Laboratories, USA), dephosphorylated active *β*-catenin (05-665, Millipore, Germany), Sirt3 (1 : 500, ab189860, Abcam, USA), matrix metallopeptidase-7 (MMP-7, 1 : 400, ab5706; Abcam, USA), and cyclooxygenase-2 (COX-2, 1 : 1000, ab62331, Abcam, USA). *α*-Tubulin was purchased from the Proteintech Company (Hubei, China). The secondary antibodies of goat antirabbit (1 : 5000, ab6721; Abcam, USA) or goat antimouse (1 : 5000, A21010; Abbkine, USA) were used. PVDF membranes were visualized using a chemiluminescence Western blotting detection reagent. The signal intensity of each immunoblot was analyzed using ImageJ software (version 1.48v; NIH, USA), and each band density was normalized by the *α*-tubulin protein expression level. To clearly present the results, the value of protein expression in the sham-operated or control group was further normalized to 1 using the mean.

### 2.9. Quantitative Real-Time PCR (qRT-PCR)

The mRNA expression was analyzed by qRT-PCR as previously described [35]. TRIzol Reagent (Invitrogen, USA) and Transcriptor First Strand cDNA Synthesis Kit (Roche, Germany) were used to extract total mRNA and synthesize cDNA, respectively. SYBR® Premix Ex Taq™ II (Takara Bio, Otsu, Shiga, Japan) was employed, and the primer sequences synthesized by Sangon (Guangzhou, China) are listed in Table 1. Samples were amplified by a Bio-Rad CFX96 Touch™ System (Bio-Rad, USA). The mRNA levels were normalized to *β*-actin and determined by the ΔΔCt method.

### 2.10. Coimmunoprecipitation (Co-IP)

Co-IP was used to analyze the interaction of VE-cadherin and *β*-catenin. The lysates of HPMECs or lung tissues were pretreated with protein A/G (GE Healthcare, USA) at 4°C for 1 h. The supernatant was incubated with anti-*β*-catenin (1 : 100) antibody overnight at 4°C and then immunoprecipitated by protein A/G overnight at 4°C. After washing six times, the complexes were analyzed by Western blot and the total protein was used as the input.

### 2.11. TCF/LEF Reporter Assay

TCF/LEF reporter assay was carried out by the procedures described previously [13]. The Cignal TCF/LEF reporter assay kit (336841; Qiagen, USA) was used according to the manufacturer's protocol. The p(GAGA)12-luc and pGL3-basic were transfected into HPMECs using Lipofectamine 3000 (Invitrogen, USA), and the medium was removed after 24 h culture. The fluorescence was analyzed using the dual-luciferase reporter assay system (E1910; Promega, USA).

### 2.12. Chromatin Immunoprecipitation Assay (ChIP)

ChIP assay was performed using the Pierce Agarose ChIP Kit (26156, New York, NY, USA) according to the manufacturer's instructions. The DNA/protein complexes were crosslinked in 1% formaldehyde (28906, NY, USA) for 10 min. The chromatin in the nuclei obtained from cells or tissues were digested by micrococcal nuclease at 4°C for 5 min. The lysates were incubated with 3 *μ*g anti-*β*-catenin antibody at 4°C overnight, and the immunoprecipitation incubated with normal rabbit IgG was used as a control. qRT-PCR was used to amplify MMP-7, and the primer sequence is listed in Table 1. Fold enrichment relative to the IgG antibody control (negative control) set to 1.0 was calculated using the ΔΔCt method.

### 2.13. Proximity Ligation Assay (PLA)

PLA was carried out using the Duolink® In Situ PLA Kit (DUO92102, Sigma-Aldrich, USA) according to the manufacturer's protocol as previously described [36]. Briefly, HPMECs cultured on coverslips were fixed with 3 : 1 acetone methanol at -20°C for 5 min and blocked with blocking solution for 30 min. Then, HPMECs were incubated with anti-VE-cadherin and anti-*β*-catenin antibodies at 4°C overnight and incubated with PLA probes at room temperature for 1 h and then at 37°C for 2 h. The amplifications were performed at 37°C, and HPMECs were then stained by DAPI for 5 min. The cells were examined by a laser-scanning confocal microscope (Leica Microsystems, Germany). To clearly present the results, the value of protein expression in the control group was further normalized to 1 using the mean.

### 2.14. Statistics

The results were expressed as mean ± SE, and GraphPad Prism software version 6.0 (San Diego, CA, USA) was employed for statistical analysis. Statistical analysis for two groups employed by Student's *t*-test; statistical analysis for three or more groups was performed using one-way analysis of variance (ANOVA) followed by Dunnett's post hoc test when *F* achieved *p* < 0.05 and there was no significant variance in homogeneity. To avoid unwanted sources of variation, results were normalized to control. *p* < 0.05 was considered significant. The number of replicates was 6 per group for each data set.

## 3. Results

### 3.1. Sepsis Damaged Microvascular Endothelial Adherens Junction Integrity and Induced Inflammatory Injury in Lung

**Figure 1:**
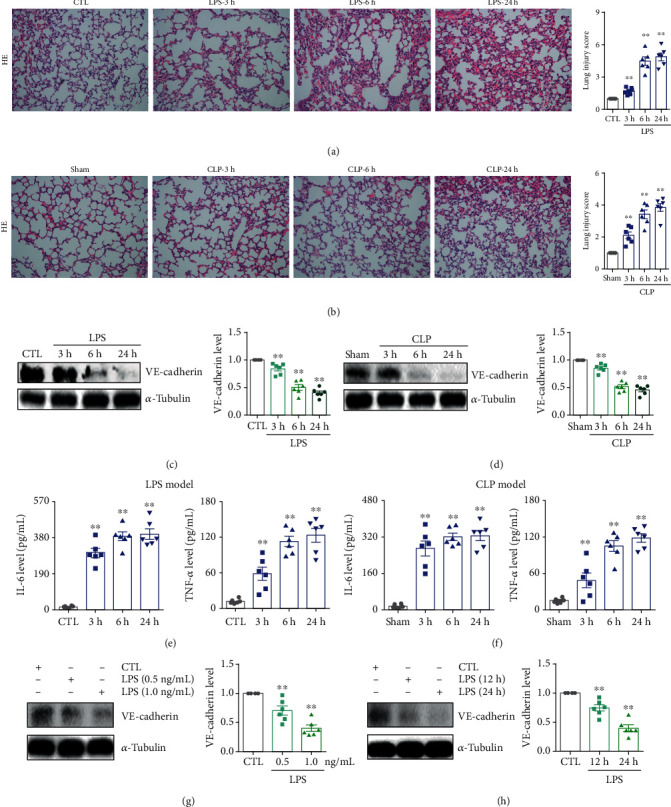
The downregulation of microvascular endothelial adherens junction protein VE-cadherin was accompanied by increased inflammation. (a) HE staining of lung tissues and lung injury scores of the LPS-induced ALI mouse model at different times. Magnification: ×200. (b) HE staining of lung tissues and lung injury scores of the CLP-induced ALI mouse model at different times. Magnification: ×200. (c) The protein expression and relative quantitative data of VE-cadherin in the LPS-induced ALI mouse model. (d) The protein expression and relative quantitative data of VE-cadherin in the CLP-induced ALI mouse model. (e) The serum IL-6 and TNF-*α* levels in the LPS-induced ALI mouse model. (f) The serum IL-6 and TNF-*α* levels in the CLP-induced ALI mouse model. (g) The protein expression and relative quantitative data of VE-cadherin in HPMECs after 24 h stimulation by LPS. (h) The protein expression and relative quantitative data of VE-cadherin in HPMECs after 1.0 ng/mL LPS stimulation. ^∗∗^*p* < 0.01 compared with the CTL or sham-operated group (*n* = 6). Dot presents the single data results in the bar graph.

To investigate the role of Sirt3 in maintaining lung microvascular endothelial barrier integrity, we first established LPS- and CLP-induced ALI mouse models. As shown in Figure 1(a), HE staining results indicated that compared with the control group, LPS caused the extensive accumulation of neutrophils and cellular debris in lung tissue in a time-dependent manner. Similar results were also found in the CLP mouse model. As shown in Figure 1(b), compared with the sham-operated group, CLP surgery induced lung neutrophil infiltration and cellular debris deposition in a time-dependent manner. These results demonstrated the successful establishment of ALI models. Since the adherens junctional integrity plays an important role in maintaining lung function and preventing inflammatory injury [37], we next determined the protein expression of VE-cadherin, an important microvascular endothelial adherens junction protein. As shown in Figures 1(c) and 1(d), both LPS- and CLP-induced ALI were accompanied by the remarkable downregulation of VE-cadherin protein in a time-dependent manner. In addition, compared with the control group, serum IL-6 and TNF-*α* levels in the LPS group were significantly elevated in a time-dependent manner (Figure 1(e)). Similar results were also observed in the CLP-induced ALI model (Figure 1(f)), indicating the breakdown of the lung microvascular endothelial adherens junction involved in the lung inflammatory injury, which was consistent with previous studies [37, 38]. Next, we examined the VE-cadherin protein expression in LPS-stimulated HPMECs. As shown in Figures 1(g) and 1(h), LPS stimulation resulted in the downregulation of VE-cadherin protein in dose- and time-dependent manners, which confirmed that the breakdown of the lung microvascular barrier was related to the lung inflammatory injury.

### 3.2. The Upregulation of *β*-Catenin Activity Was Accompanied by Increased Inflammatory Injury and Damaged Microvascular Endothelial Adherens Junction

**Figure 2:**
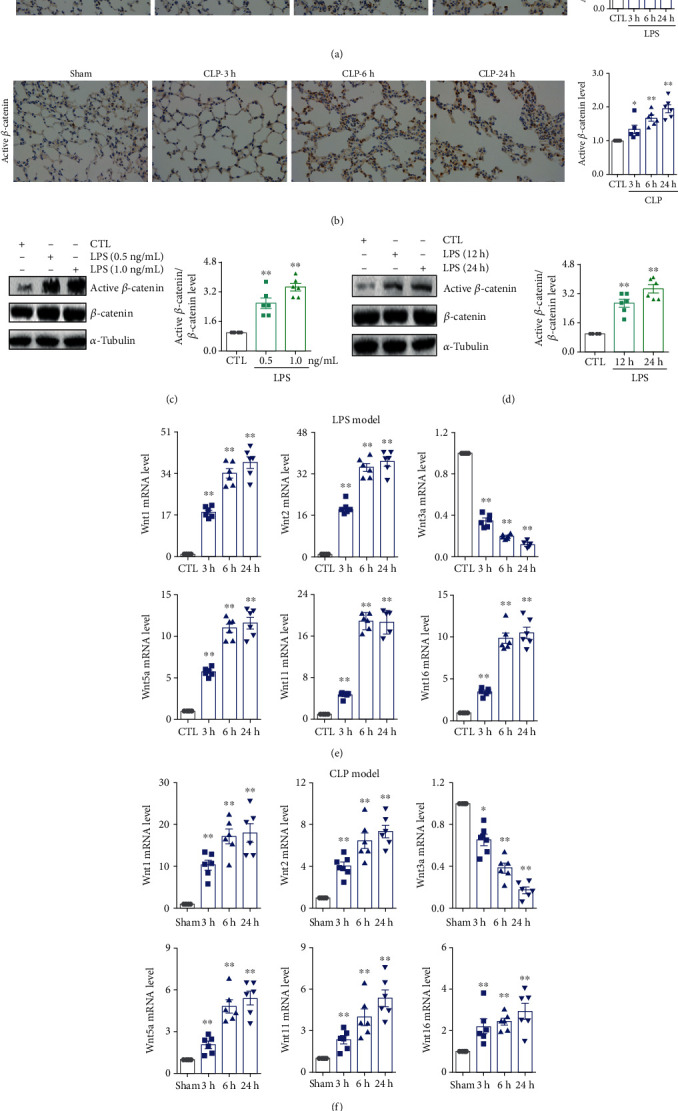
*β*-Catenin was activated during sepsis-induced ALI. (a) Immunohistochemical staining and relative quantitative data of active *β*-catenin in lung tissues of the LPS-induced ALI mouse model at different times. Magnification: ×400. (b) Immunohistochemical staining and relative quantitative data of active *β*-catenin in lung tissues of the CLP-induced ALI mouse model at different times. Magnification: ×400. (c) The protein expression and relative quantitative data of active *β*-catenin in HPMECs after 24 h stimulation by LPS. (d) The protein expression and relative quantitative data of active *β*-catenin in HPMECs after 1.0 ng/mL LPS stimulation. (e) The mRNA levels of Wnt1, Wnt2, Wnt3a, Wnt5a, Wnt11, and Wnt16 in lung tissues of the LPS-induced ALI mouse model at different times. (f) The mRNA levels of Wnt1, Wnt2, Wnt3a, Wnt5a, Wnt11, and Wnt16 in lung tissues of the CLP-induced ALI mouse model at different times. ^∗^*p* < 0.05 and ^∗∗^*p* < 0.01 compared with the CTL or sham-operated group (*n* = 6). Dot presents the single data results in the bar graph.

The interaction of *β*-catenin and VE-cadherin maintains the adherens junctional integrity, while the breakdown of the adherens junction releases *β*-catenin from the cytomembrane [8]. Therefore, we next examined whether *β*-catenin was activated during ALI. As shown in Figures 2(a) and 2(b), compared with the control or the sham-operated group, the expression of active *β*-catenin protein significantly increased in the nucleus of lung tissues in LPS- and CLP-induced ALI models in a time-dependent manner, indicating the activation of *β*-catenin during ALI. As shown in Figures 2(c) and 2(d), LPS stimulation also promoted *β*-catenin activity in HPMECs in dose- and time-dependent manners, which confirmed the positive correlation between *β*-catenin activity and ALI. Since the activation of *β*-catenin contributes to the destruction of the microvascular adherens junction directly through increasing downstream gene MMP-7 expression to damage the adherens junction [37, 39, 40], we next examined the expression of the ligand of *β*-catenin, the Wnt family. As shown in Figure 2(e), compared with the control group, the mRNA expressions of Wnt1, Wnt2, Wnt5a, Wnt11, and Wnt16 were significantly upregulated, while the mRNA expression of Wnt3a was significantly downregulated in the LPS group in a time-dependent manner, which also indicated the activation of *β*-catenin. And these results were consistent with previous studies [40, 41]. In the CLP-induced ALI mouse model, compared with sham-operated group, the mRNA expressions of Wnt1, Wnt2, Wnt5a, Wnt11, and Wnt16 increased substantially, and the mRNA expression of Wnt3a decreased in a time-dependent manner. These results demonstrated that the activation of *β*-catenin during ALI was accompanied by inflammation and damaged microvascular endothelial adherens junction.

### 3.3. The Weakened Interaction of VE-Cadherin and *β*-Catenin Enhanced *β*-Catenin Transcriptional Activity during ALI

**Figure 3:**
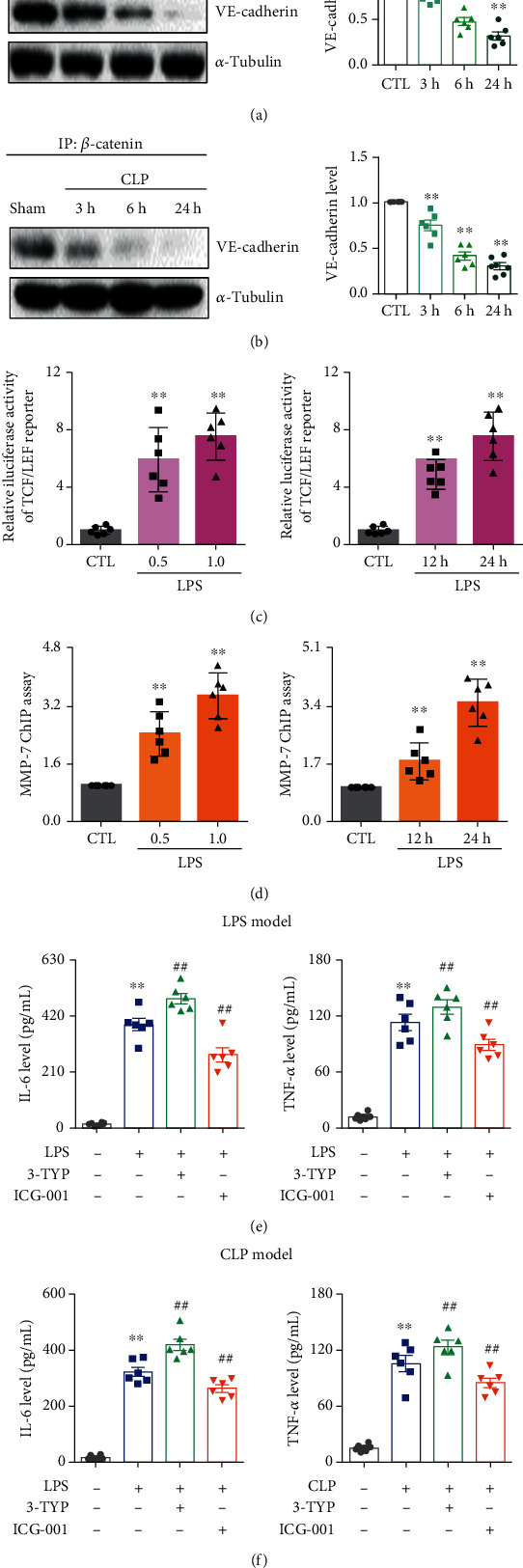
The dissociation of VE-cadherin/*β*-catenin complex and the activation of *β*-catenin contributed to microvascular endothelial adherens junction dysfunction and inflammation. (a) The protein expression and relative quantitative data of VE-cadherin in lung tissues of LPS-induced ALI mouse model at different times. (b) The protein expression and relative quantitative data of VE-cadherin in lung tissues of CLP-induced ALI mouse model at different times. (c) The luciferase activity of TCF/LEF reporter in HPMECs after 24 h stimulation by LPS or 1.0 ng/mL LPS stimulation, respectively. (d) ChIP assay results of MMP-7 in HPMECs after 24 h stimulation by LPS or 1.0 ng/mL LPS stimulation. (e) The serum IL-6 and TNF-*α* levels in the LPS-induced ALI mouse model after treatment with 3-TYP or ICG-001. (f) The serum IL-6 and TNF-*α* levels in the CLP-induced ALI mouse model after treatment with 3-TYP or ICG-001. ^∗∗^*p* < 0.01 compared with the CTL- or sham-operated group (*n* = 6). ^##^*p* < 0.01 compared with the LPS or CLP group (*n* = 6). Dot presents the single data results in the bar graph.

Next, the interactions of VE-cadherin and *β*-catenin were investigated using the Co-IP method. As shown in Figures 3(a) and 3(b), compared with the control or the sham-operated group, the interaction of VE-cadherin and *β*-catenin obviously lowered after LPS treatment or CLP surgery in a time-dependent manner. The weakened interaction of VE-cadherin and *β*-catenin may contribute to *β*-catenin accumulation in the cytoplasm and translocation into the nucleus to further trigger its transcriptional activity of MMP-7. As shown in Figure 3(c), compared with the control group, LPS stimulation promoted luciferase activity of TCF/LEF activity in dose- and time-dependent manners, indicating the activation of *β*-catenin in HPMECs during lung inflammation. LPS stimulation also resulted in the significant upregulation of the MMP-7 promoter (Figure 3(d)). These results elucidated that ALI may induce inflammation through promoting *β*-catenin activation that further increased MMP-7 expression to aggravate the breakdown of the microvascular adherens junction. Our further experiments also confirmed these hypotheses. Treatment with the *β*-catenin inhibitor ICG-001 significantly decreased serum IL-6 and TNF-*α* levels (Figures 3(e) and 3(f)), indicating that *β*-catenin activation was positively related to inflammation in ALI. Notably, treatment with the Sirt3 inhibitor 3-TYP significantly increased serum inflammatory factor levels in LPS- and CLP-induced ALI models, indicating that Sirt3 was negatively related to lung inflammation.

### 3.4. The Downregulation of Sirt3 Is Accompanied by Lowered Microvascular Adherens Junction Integrity and Elevated Inflammation

**Figure 4:**
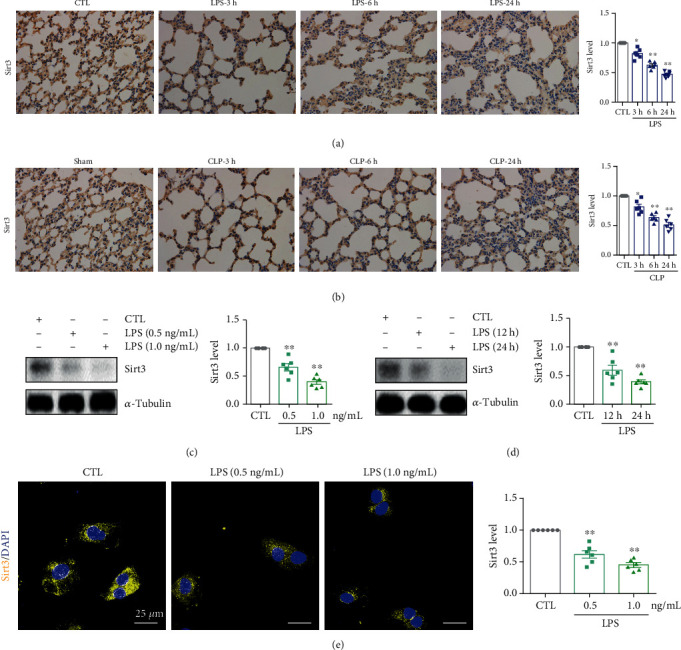
The downregulation of Sirt3 was accompanied with increased lung inflammation during ALI. (a) Immunohistochemical staining and relative quantitative data of Sirt3 in lung tissues of the LPS-induced ALI mouse model at different times. Magnification: ×400. (b) Immunohistochemical staining and relative quantitative data of Sirt3 in lung tissues of the CLP-induced ALI mouse model at different times. Magnification: ×400. (c) The protein expression and relative quantitative data of Sirt3 in HPMECs after 24 h stimulation by LPS. (d) The protein expression and relative quantitative data of Sirt3 in HPMECs after 1.0 ng/mL LPS stimulation. (e) Immunofluorescence staining and relative quantitative data of Sirt3 after 24 h stimulation by LPS. ^∗^*p* < 0.05 and ^∗∗^*p* < 0.01 compared with the CTL- or sham-operated group (*n* = 6). Dot presents the single data results in the bar graph.

Although we determined the relationship between inflammatory injury and Sirt3, the expression of Sirt3 during ALI remained unclear. Hence, we investigated the expression of Sirt3 protein in ALI mouse models and HPMECs. As shown in Figure 4(a), compared with the control group, Sirt3 expression significantly declined in the LPS group in a time-dependent manner. Similar results were found in the CLP model. Compared with the sham-operated group, Sirt3 protein expression was significantly downregulated in a time-dependent manner after CLP surgery (Figure 4(b)). To address Sirt3 expression in lung microvascular endothelial cells, HPMECs were employed. LPS stimulation resulted in the remarkable downregulation of Sirt3 protein in dose- and time-dependent manners (Figures 4(c) and 4(d)). Immunofluorescent staining of the Sirt3 protein also confirmed that the downregulation of Sirt3 was accompanied by lowered adherens junction integrity during lung inflammation (Figure 4(e)).

### 3.5. Sirt3 Promoted Microvascular Endothelial Interaction of VE-Cadherin and *β*-Catenin to Maintain Endothelial Adherens Junction Integrity

**Figure 5:**
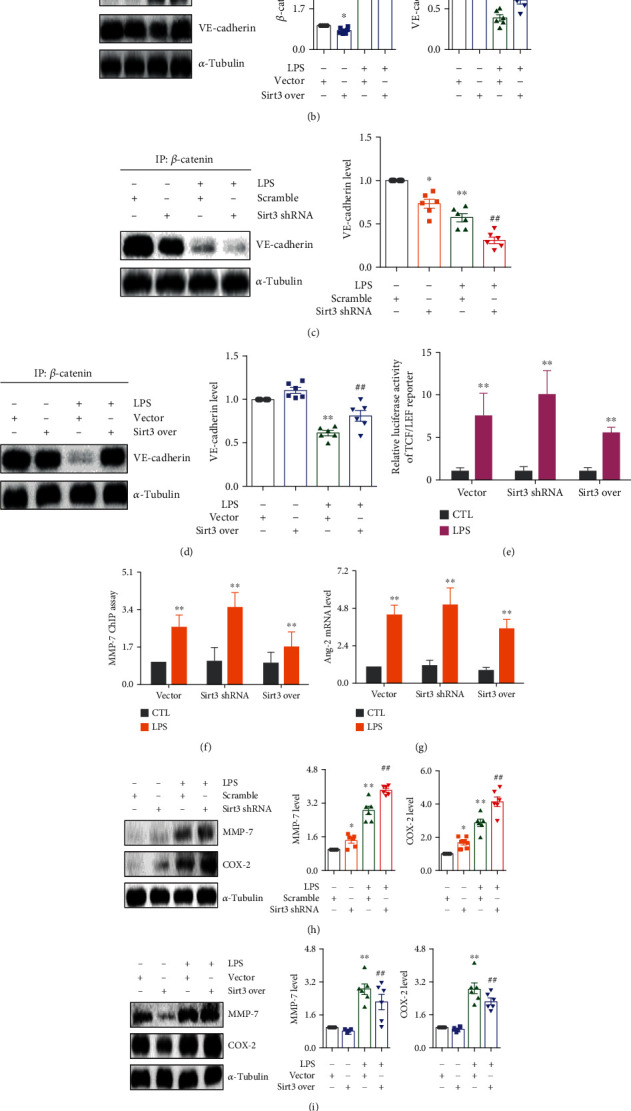
Sirt3 enhanced the stability of VE-cadherin/*β*-catenin complex and inhibited *β*-catenin transcriptional activity to maintain microvascular endothelial adherens junction integrity. (a) The protein expression and relative quantitative data of VE-cadherin and *β*-catenin in HPMECs after transfection with scramble or Sirt3 shRNA. (b) The protein expression and relative quantitative data of VE-cadherin and *β*-catenin in HPMECs after transfection with vector or Sirt3 over. (c) The protein expression and relative quantitative data of VE-cadherin in HPMECs after transfection with scramble or Sirt3 shRNA. (d) The protein expression and relative quantitative data of VE-cadherin in HPMECs after transfection with vector or Sirt3 over. (e) The luciferase activity of TCF/LEF reporter in HPMECs after transfection with Sirt3 shRNA or Sirt3 over. (f) ChIP assay results of MMP-7 in HPMECs transfected with Sirt3 shRNA or Sirt3 over after 24 h stimulation. (g) The mRNA levels of Ang-2 in HPMECs transfected with Sirt3 shRNA or Sirt3 over after 24 h stimulation. (h) The protein expression and relative quantitative data of MMP-7 and COX-2 in HPMECs after transfection with scramble or Sirt3 shRNA. (i) The protein expression and relative quantitative data of MMP-7 and COX-2 in HPMECs after transfection with vector or Sirt3 over. (j) Immunofluorescence staining and relative quantitative data of PLA signals between VE-cadherin and *β*-catenin in HPMECs transfected with Sirt3 shRNA or Sirt3 over after 24 h stimulation by LPS. ^∗^*p* < 0.05 and ^∗∗^*p* < 0.01 compared with the CTL- or sham-operated group (*n* = 6). ^##^*p* < 0.01 compared with the LPS or CLP group (*n* = 6). Dot presents the single data results in the bar graph.

To decipher the role of Sirt3 in the interaction of VE-cadherin and *β*-catenin, plasmids expressing Sirt3 shRNA, scramble, Sirt3 over, and vector were employed. As shown in Figure S1, the expression of the Sirt3 protein significantly decreased in HPMECs after transfection with Sirt3 shRNA, while the expression of the Sirt3 protein significantly increased after transfection with Sirt3 over. We first examined the effects of Sirt3 on *β*-catenin and VE-cadherin expression. As shown in Figure 5(a), compared with the scramble-transfected LPS group, Sirt3 deficiency remarkably amplified LPS-induced *β*-catenin upregulation and VE-cadherin downregulation after being transfected with the Sirt3 shRNA plasmid, while Sirt3 overexpression by the Sirt3 over plasmid inhibited *β*-catenin protein expression and promoted VE-cadherin protein expression in HPMECs (Figures 5(a) and 5(b)). Co-IP results elucidated that Sirt3 also affected the interaction of *β*-catenin and the VE-cadherin protein. As shown in Figures 5(c) and 5(d), LPS stimulation suppressed the interaction of *β*-catenin and the VE-cadherin protein in HPMECs. Sirt3 deficiency weakened their interaction while Sirt3 overexpression strengthened their interaction (Figures 5(c) and 5(d)).

We further investigated the effects of Sirt3 on *β*-catenin transcriptional activity and Ang-2 expression that contributes to the breakdown of the microvascular endothelial adherens junction. While Sirt3 deficiency elevated the luciferase activity of the TCF/LEF reporter induced by LPS, Sirt3 overexpression suppressed the luciferase activity of the TCF/LEF reporter (Figure 5(e)), indicating the regulatory effects of Sirt3 on *β*-catenin transcriptional activity. Additionally, Sirt3 deficiency increased the expression of MMP-7 promoters and Ang-2 mRNA expression, while Sirt3 overexpression decreased the expression of MMP-7 promoters and Ang-2 mRNA expression in HPMECs (Figures 5(f) and 5(g)). Furthermore, knockdown of Sirt3 facilitated the upregulation of the MMP-7 and COX-2 protein expression induced by LPS, while overexpression of Sirt3 inhibited their protein expression (Figures 5(h) and 5(i)). We also observed interaction between endogenous *β*-catenin and VE-cadherin in the cytomembrane using in situ PLA. Compared with the control group, distinct *β*-catenin-VE-cadherin signals were lowered upon LPS stimulation, indicating the weakened interaction of *β*-catenin and the VE-cadherin protein by LPS stimulation (Figure 5(j)). Of note, Sirt3 deficiency aggravated their separation, while Sirt3 overexpression promoted their interaction (Figure 5(j)). Taken together, these results demonstrated that Sirt3 modulated the microvascular endothelial adherens junction integrity to inhibit lung inflammation through maintaining the interaction of *β*-catenin and the VE-cadherin protein *in vitro*.

### 3.6. Sirt3 Suppressed Lung Inflammation by Maintaining the Microvascular Endothelial Adherens Junction Integrity

**Figure 6:**
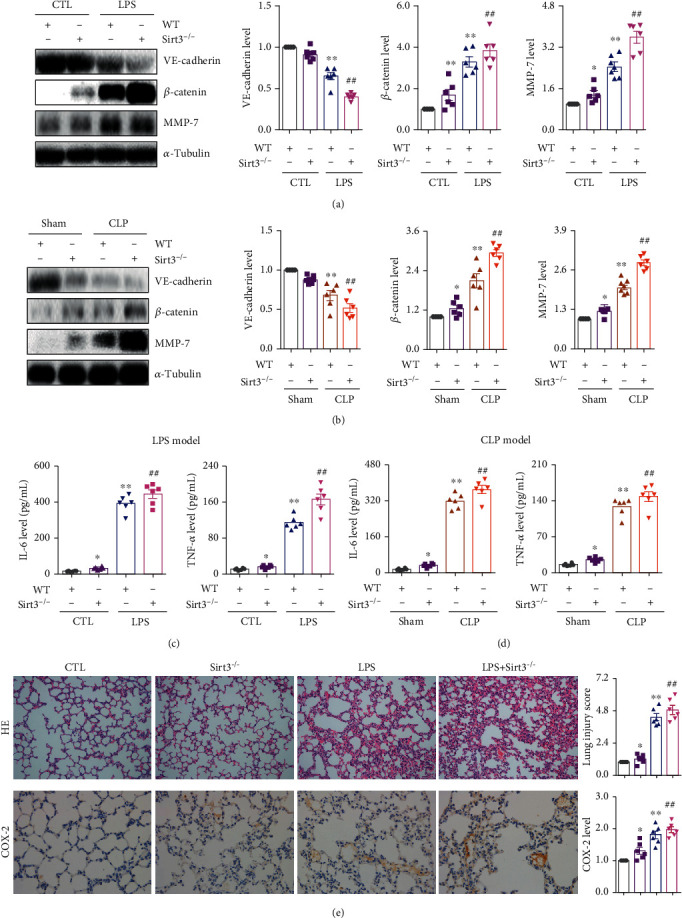
Sirt3-mediated VE-cadherin/*β*-catenin complex maintained microvascular endothelial adherens junction integrity to suppress inflammation. (a) The protein expression and relative quantitative data of VE-cadherin, *β*-catenin, and MMP-7 in LPS-induced ALI WT and Sirt3^−/−^ mouse model at 6 h. (b) The protein expression and relative quantitative data of VE-cadherin, *β*-catenin, and MMP-7 in CLP-induced ALI WT and Sirt3^−/−^ mouse model at 6 h. (c) The serum IL-6 and TNF-*α* levels in LPS-induced ALI WT and Sirt3^−/−^ mouse model at 6 h. (d) The serum IL-6 and TNF-*α* levels in CLP-induced ALI WT and Sirt3^−/−^ mouse model at 6 h. (e) HE staining, immunohistochemical staining, and relative quantitative data of COX-2 in lung tissues of LPS-induced ALI WT and Sirt3^−/−^ mouse model at 6 h. Magnification: ×200; HE. Magnification: ×400; COX-2. ^∗^*p* < 0.05 and ^∗∗^*p* < 0.01 compared with the CTL- or sham-operated group (*n* = 6). ^##^*p* < 0.01 compared with the LPS or CLP group (*n* = 6). Dot presents the single data results in the bar graph. WT: wild type.

**Figure 7:**
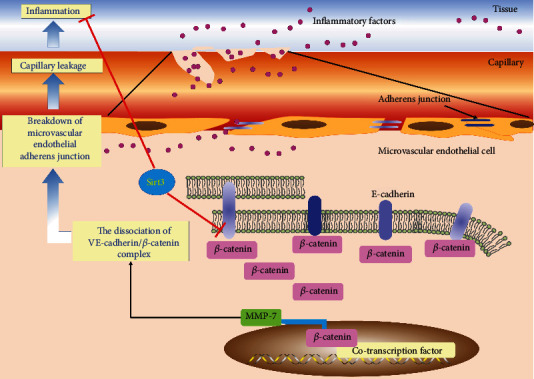
Sirt3 maintained microvascular endothelial adherens junction integrity to attenuate lung inflammation by acting on the stability of VE-cadherin/*β*-catenin complex. Sepsis induced VE-cadherin downregulation, *β*-catenin activation, and, importantly, the dissociation of VE-cadherin/*β*-catenin complex in lung microvascular endothelial cells and ALI animal models. These damaged adherens junctions and triggered the *β*-catenin-mediated MMP-7 expression to further destroy VE-cadherin/*β*-catenin complex, which eventually resulted in the breakdown of microvascular endothelial adherens junction. These events facilitated the transfer of inflammatory factor through microvascular endothelial cells into the capillary and contributed to capillary leakage and ultimately lung inflammation. Notably, we first found that Sirt3 could inhibit inflammation through maintaining microvascular endothelial adherens junction integrity by acting on the interaction of VE-cadherin and *β*-catenin.

To confirm the role of Sirt3 on the microvascular endothelial adherens junction integrity to inhibit lung inflammation, we used the Sirt3^−/−^ mouse model. As shown in Figure 6(a), compared with the LPS group in the wild type mouse, the protein expression of VE-cadherin significantly decreased, while the protein expression of *β*-catenin and MMP-7 significantly increased in the Sirt3^−/−^ mouse. Similar results were also found in the CLP model. Compared with the wild type mouse, the downregulation of VE-cadherin and the upregulation of *β*-catenin and MMP-7 were observed in the Sirt3^−/−^ mouse after CLP surgery (Figure 6(b)). In addition, compared with the control or the sham-operated group, serum IL-6 and TNF-*α* levels in the Sirt3^−/−^ mouse were higher than those in the wild type mouse in the LPS- or CLP-induced group (Figures 6(c) and 6(d)). These results demonstrated that Sirt3 modulated the microvascular endothelial adherens junction integrity to inhibit lung inflammation *in vivo*. HE staining and immunohistochemical staining of COX-2 also showed that lung inflammation in the Sirt3^−/−^ mouse was worse than that in the wild type mouse (Figure 6(e)). These results confirmed that Sirt3 modulated the microvascular endothelial adherens junction integrity to inhibit lung inflammation by acting on the interaction of *β*-catenin and the VE-cadherin protein (Figure 7).

## 4. Discussion

Sepsis leads to high mortality and morbidity due to multiple organ dysfunction including ARDS/ALI that causes severe systemic inflammatory response syndrome. The microvascular endothelial barrier dysfunction, microvascular permeability imbalance, and capillary leakage induced by sepsis play important roles in the occurrence and development of organ dysfunction [42, 43]. The microvascular endothelial barrier consists of single-layer continuous endothelial cells between the blood and interstitial tissue that controls many physiological roles including the transportation of water and nutrients, the tension of blood vessels, the aggregation and adhesion of inflammatory mediators, and hemostasis and thrombosis. The adherens junction between microvascular endothelial cells maintains microvascular endothelial barrier function and modulates its stability and permeability, while the breakdown of the adherens junction causes microvascular endothelial barrier dysfunction, thus resulting in the increased microvascular endothelial permeability and leakage [44, 45]. During sepsis-induced ARDS/ALI, the systemic inflammatory response is rapidly activated, and a fast and effective defense mechanism is triggered to alleviate microvascular endothelial barrier permeability that further contributes to the production and migration of monocytes, macrophages, and other immune cells to resist pathogenic microorganisms and endotoxin invasion. Meanwhile, sepsis leads to the release of excessive inflammatory mediators that results in the imbalance of microvascular endothelial barrier permeability, the aggravation of microvascular leakage, and, ultimately, circulatory failure and multiple organ dysfunction. The interaction among the microvascular endothelial barrier, the adherens junction, and excessive inflammatory mediators plays a significant role in the pathophysiology of sepsis-induced ARDS/ALI, which suggests that the protection of the microvascular endothelial adherens junction may serve as the novel therapeutic target and strategy to prevent ARDS/ALI.

In the present study, we identified Sirt3 as a protective factor for the adherens junction of the lung microvascular endothelial barrier. Sirt3, the main mitochondrial molecule in the Sirt family, alleviated ARDS/ALI through regulating cellular bioenergetics, mitochondrial metabolism, and inflammatory responses [25, 46, 47]. However, few studies investigated the effect and mechanism of Sirt3 on the adherens junction of the lung microvascular endothelial barrier to inhibit inflammation. Here, we found that in LPS- and CLP-induced ALI mouse models, the protein expression of Sirt3 declined in a time-dependent manner that was accompanied by the increased serum IL-6 and TNF-*α* levels, indicating the occurrence of systemic inflammation. Pharmacological inhibition of Sirt3 by 3-TYP treatment or deletion of Sirt3 by gene editing technology significantly amplified systemic inflammation *in vivo*. These results indicated the effects of Sirt3 against inflammation in ALI. Additional experiments elucidated that Sirt3 maintained the microvascular endothelial adherens junction in HPMECs. Sirt3 overexpression significantly protected the adherens junction of the lung microvascular endothelial barrier in HPMECs, while Sirt3 deficiency promoted the breakdown of the adherens junction of the lung microvascular endothelial barrier. These results confirmed the protective role of Sirt3 on maintaining the adherens junction of the lung microvascular endothelial barrier to attenuate inflammation, which first revealed that Sirt3 suppressed ARDS/ALI-induced inflammation through maintaining the adherens junction of the lung microvascular endothelial barrier.

Furthermore, we explored the underlying mechanism of Sirt3 on maintaining the adherens junction of the lung microvascular endothelial barrier. The integrity of the adherens junction involves the interaction between VE-cadherin and *β*-catenin. The dimerization of VE-cadherin occurs in the cytomembrane, and the dimerization of the adjacent cellular VE-cadherin dimer forms cadherin clusters. The cytoplasmic domain of VE-cadherin contains the binding sites of the VE-cadherin/*β*-catenin complex. In the physiological status, VE-cadherin and *β*-catenin existed as a complex and suppressed *β*-catenin transcriptional activity. However, once microvascular endothelial cells were injured, the VE-cadherin/*β*-catenin complex dissociated and the VE-cadherin-dominant intercellular adherens junction became damaged, which caused *β*-catenin accumulation in the cytoplasm and translocation into the nucleus to initiate *β*-catenin transcriptional activity [48].

In the present study, we found that the protein expression of Sirt3 was positively related to VE-cadherin expression but negatively related to *β*-catenin activity in the ALI model. Sirt3 overexpression strengthened the interaction of VE-cadherin and *β*-catenin, while Sirt3 deficiency weakened their interaction. Additionally, Sirt3 also regulated *β*-catenin transcriptional activity. Accompanied by *β*-catenin released from the complex, sepsis-induced ALI resulted in the upregulation of Wnt ligands that further accumulated *β*-catenin in the cytoplasm and translocated into the nucleus, all of which promoted *β*-catenin transcriptional activity. MMP-7, the downstream target gene of *β*-catenin, participates in the breakdown of the intercellular adherens junction that ultimately results in microvascular leakage and inflammation aggravation [48]. Here, the upregulations of MMP-7 were detected during lung inflammation *in vivo* and *in vitro*. Sirt3 also induced Ang-2 expression to damage the lung microvascular endothelial barrier. These results indicated the underlying mechanism of Sirt3 on maintaining the adherens junction of the lung microvascular endothelial barrier by acting on the interaction of VE-cadherin and *β*-catenin (Figure 7).

## 5. Conclusion

Although Sirt3 is known to modulate metabolism to suppress inflammation in ARDS/ALI, we first focus Sirt3 on the microvascular endothelial barrier *via* the VE-cadherin/*β*-catenin complex. We identified for the first time Sirt3 as the crucial mediator of sepsis-induced lung microvascular endothelial barrier integrity. In summary, the present study provides novel insights into the mechanism of Sirt3 against lung inflammatory injury. We identified Sirt3 as a modulator of the adherens junction in the lung microvascular endothelial barrier to suppress inflammation through regulating the interaction of VE-cadherin and *β*-catenin. These results provide the potential therapeutic target and novel strategy to prevent inflammation in ARDS/ALI.

## Figures and Tables

**Table 1:** Nucleotide sequences of the primers used for qRT-PCR and ChIP.

Gene	Forward	Reverse	Product size (bp)
Primers used for qRT-PCR			
Wnt1	CTGGCTGGGTTTCTGCTAC	GAGGAGGCTACGTTCACAATAC	106
Wnt2	CTCCTCAGCTGGAGTTGTATTT	GGCGCTTCCCATCTTCTT	94
Wnt3a	TGTTGGGCCACAGTATTCC	GGCATGATCTCCACGTAGTT	111
Wnt5a	CCTTCGCCCAGGTTGTAAT	AGAGAGGCTGTGCTCCTATAA	102
Wnt11	AACAGGATCCCAAGCCAATAA	CCATGGCACTTACACTTCATTTC	99
Wnt16	GGTTCAGCAGAAAGTTCCTAGA	GCCTTCCAGCTTCATTGTTATG	102
GAPDH	GGTGTGAACCATGAGAAGTATGA	GAGTCCTTCCACGATACCAAAG	123
Primers used for ChIP			
MMP-7	GGAGACCCAAAGAAGGGAATTA	TGCTGTGTGGCTGGATTAG	105

## Data Availability

The data used to support the findings of this study are available from the corresponding authors upon request.

## Supplementary Materials

Supplementary Materials Figure S1: the protein expression of Sirt3 *in vitro* or *in vivo*. (a) The protein expression of Sirt3 in HPMECs after transfection with the plasmid expressing shRNA against human Sirt3 (Sirt3 shRNA) or the plasmid containing scramble (scramble). (b) The protein expression of Sirt3 in HPMECs after transfection with the plasmid expressing full-length human Sirt3 cDNA (Sirt3 over) or the plasmid containing empty plasmids (vector). (c) The protein expression of Sirt3 from lung tissues of WT or Sirt3^−/−^ mice.
