# Supplementary material for: Sirt3 Maintains Microvascular Endothelial Adherens Junction Integrity to Alleviate Sepsis-Induced Lung Inflammation by Modulating the Interaction of VE-Cadherin and β-Catenin
Source: Oxid Med Cell Longev. 2021 Oct 1;2021:8978795. doi: 10.1155/2021/8978795 (PMC8500765; doi:10.1155/2021/8978795)
Supplement: Supplementary Materials — Figure S1: the protein expression of Sirt3 in vitro or in vivo. (a) The protein expression of Sirt3 in HPMECs after transfection with the plasmid expressing shRNA against human Sirt3 (Sirt3 shRNA) or the plasmid containing scramble (scramble). (b) The protein expression of Sirt3 in HPMECs after transfection with the plasmid expressing full-length human Sirt3 cDNA (Sirt3 over) or the plasmid containing empty plasmids (vector). (c) The protein expression of Sirt3 from lung tissues of WT or Sirt3−/− mice. [file 8978795.f1.docx]

**Supplementary Materials**


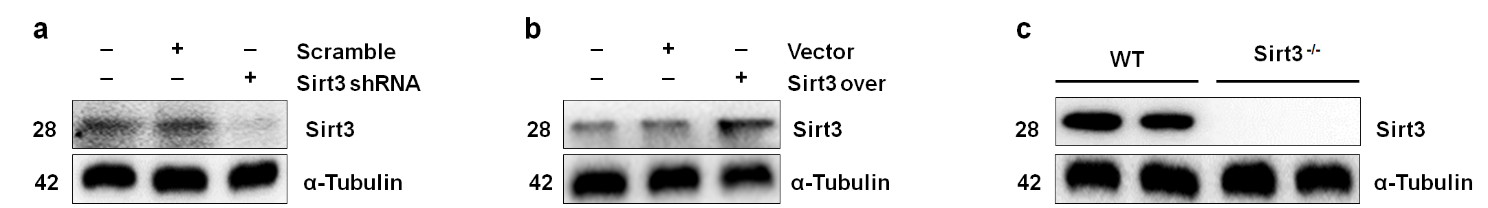


**Figure S1. The protein expression of Sirt3 *in vitro* or *in vivo*.** (a) The protein expression of Sirt3 in HPMECs after transfection with the plasmid expressing shRNA against human Sirt3 (Sirt3 shRNA) or the plasmid containing scramble (scramble). (b) The protein expression of Sirt3 in HPMECs after transfection with the plasmid expressing full-length human Sirt3 cDNA (Sirt3 over) or the plasmid containing empty plasmids (vector). (c) The protein expression of Sirt3 from lung tissues of WT or Sirt3^-/-^ mice.
